# Application of failure mode and effects analysis combined with bundled management measures in reducing ICU-acquired infections

**DOI:** 10.3389/fmed.2026.1801802

**Published:** 2026-05-22

**Authors:** Meng Zhang, Tuluke Ku, Huimin Jia, Yan Wang, Jiawang Zhou, Weihao Zhang

**Affiliations:** 1 School of Public Health, Xinjiang Medical University, Ürümqi, China; 2 College of Medical Engineering Technology, Xinjiang Medical University, Ürümqi, China; 3 The Affiliated Cancer Hospital of Xinjiang Medical University, Ürümqi, China

**Keywords:** bundled management, failure mode and effects analysis, hospital infection control, intensive care unit, risk management

## Abstract

**Objective:**

To systematically evaluate the application value and implementation effectiveness of a Failure Mode and Effects Analysis (FMEA)-based bundled management strategy in the precise prevention and control of hospital-acquired infections (HAIs) in the Intensive Care Unit (ICU) of a specialized oncology hospital.

**Methods:**

A single-cohort study with pre-post measurements was conducted in the ICU of a tertiary Grade A specialized oncology hospital. The pre-implementation period included 2,975 patients admitted from January to December 2024, receiving conventional HAI prevention and control measures. The post-implementation period included 1,522 patients admitted from January to June 2025, who received an FMEA-driven bundled management strategy in addition to conventional measures. A multidisciplinary FMEA team identified potential failure modes using the “Man, Machine, Material, Method, Environment, Measurement” framework. Risks were quantified using the Risk Priority Number (RPN = Severity × Occurrence × Detection), and high-risk failure modes (RPN ≥ 80) were targeted with multi-dimensional bundled interventions (e.g., multi-departmental coordination, stratified training, hand hygiene improvement). Outcomes included HAI incidence, device-associated infection rates [Ventilator-Associated Pneumonia (VAP), Central Line-Associated Bloodstream Infection (CLABSI), Catheter-Associated Urinary Tract Infection (CAUTI)], hand hygiene compliance, and environmental cleaning quality.

**Results:**

Following the intervention, overall HAI incidence decreased significantly from 2.6% (77 infections in 2,975 patients) to 1.6% (25 infections in 1,522 patients) (χ^2^ = 4.062, *P* < 0.05), a relative reduction of 36.7%. Device-associated infection rates showed notable control: CAUTI decreased from 0.57‰ to 0, VAP from 1.69‰ to 0, and CLABSI remained low (0.28–0.27‰). Hand hygiene compliance among healthcare workers increased from 72.2 to 88.9% (χ^2^ = 4.578, *P* < 0.05), an improvement of 23.1%. The 24-h removal rate of fluorescent markers for environmental cleaning assessment surged from 36.1 to 76.0%. RPN values for all six high-risk failure modes decreased substantially, with reductions ranging from 28.0 to 75.0%.

**Conclusion:**

The FMEA-based bundled management strategy effectively identifies and mitigates high-risk vulnerabilities in ICU HAI prevention and control. It significantly reduces HAI and device-associated infection rates while improving hand hygiene compliance and environmental disinfection quality. This model provides an evidence-based paradigm for precise HAI prevention and control in specialized oncology hospital ICUs and holds high potential for broader clinical application.

## 1 Introduction

Hospital-acquired infections (HAIs) represent a major public health challenge in the field of global healthcare safety. They not only significantly prolong hospital stays and increase medical costs but also impose a heavy economic and psychological burden on patients, their families, and society, severely threatening the quality of care and patient safety (1–3). The Intensive Care Unit (ICU) has become a critical battleground for HAI prevention and control due to its characteristics, including the admission of critically ill patients, frequent invasive procedures, and the concentration of immunocompromised individuals, where patients are often in severe condition with weakened immunity (4).

The patient population in the ICU of a specialized oncology hospital presents distinct particularities. Compared to general hospitals, the treatment course for oncology patients is influenced by multiple factors such as age, malnutrition, invasive procedures, radiotherapy/chemotherapy, and antibiotic overuse, making it easier for pathogens to invade. Concurrently, the long-term chronic depletion of the body’s defense system by the tumor itself makes cancer patients more susceptible to infections following radiotherapy and chemotherapy (5). The superimposition of these unique high-risk factors leads to a significantly elevated risk of HAIs in the ICU of oncology hospitals compared to general hospitals. Therefore, establishing a scientific, precise, and efficient HAI prevention and control system is crucial for the ICU in a specialized oncology hospital.

Failure Mode and Effects Analysis (FMEA) is a prospective, systematic risk assessment tool used in the design phase to predict and quantitatively evaluate various potential failure causes before they occur (6, 7). This method involves forming an FMEA team, identifying potential failure modes through brainstorming sessions, calculating the Risk Priority Number (RPN) for these potential failures (8, 9), focusing on key risk events throughout the process management, implementing targeted interventions, and evaluating their effectiveness. The FMEA method can help avoid or reduce medical errors that cause harm to patients (10). Studies have shown that FMEA plays a crucial role in the HAI prevention and control process (11–13). Currently, FMEA has been widely applied in continuous quality improvement efforts such as multidrug-resistant organism prevention and control (14), improving endoscope cleaning quality, surgical site infection prevention, and central line-associated bloodstream infection control, achieving significant results (15).

However, existing research predominantly focuses on single infection types or specific operational steps, lacking systematic risk assessment and comprehensive intervention studies targeting the entire HAI prevention and control process in the ICU of specialized oncology hospitals.

Bundled care management, as a quality improvement strategy that integrates multiple evidence-based measures for implementation, exerts synergistic effects through multi-dimensional collaborative interventions and has been widely applied in critical care medicine (16). Building on this, this study organically combines the prospective risk assessment of FMEA with bundled care management. It aims to systematically identify high-risk vulnerabilities within the HAI prevention and control process in the ICU of a specialized oncology hospital, and to develop and implement multi-dimensional, targeted bundled intervention strategies. The goal is to reduce the incidence of HAIs, improve the quality of medical care, ensure patient safety, and provide an evidence-based practice model for similar healthcare institutions.

## 2 Subjects and methods

### 2.1 Study design and subjects

A single-cohort study with pre-post measurements was conducted in the ICU of a tertiary Grade A specialized oncology hospital. Patients admitted from January to December 2024 (pre-implementation period) received conventional HAI prevention and control measures and served as the pre-implementation reference. Patients admitted from January to June 2025 (post-implementation period) received the FMEA-driven bundled management strategy in addition to conventional measures. Outcome measures were compared between the two periods within the same ICU cohort. Baseline characteristics (age, sex, primary diagnosis, APACHE II score, comorbidities, and ICU length of stay) were extracted from electronic medical records for all patients in both the pre-implementation and post-implementation periods to ensure comparability of the two independent samples.

### 2.2 Research methods

#### 2.2.1 Establishment of the FMEA team

A multidisciplinary FMEA team was established. Team members included the ICU director, head nurse, part-time infection control healthcare workers, full-time staff from the hospital infection control department, clinical pharmacists, and microbiologists. All members participated in unified training covering relevant concepts, methodological steps, application areas, and advantages of the FMEA approach. This training ensured that all members possessed proficient FMEA analysis skills, achieving homogeneity in the analysis process.

#### 2.2.2 Risk identification and indicator determination

**FIGURE 1:**
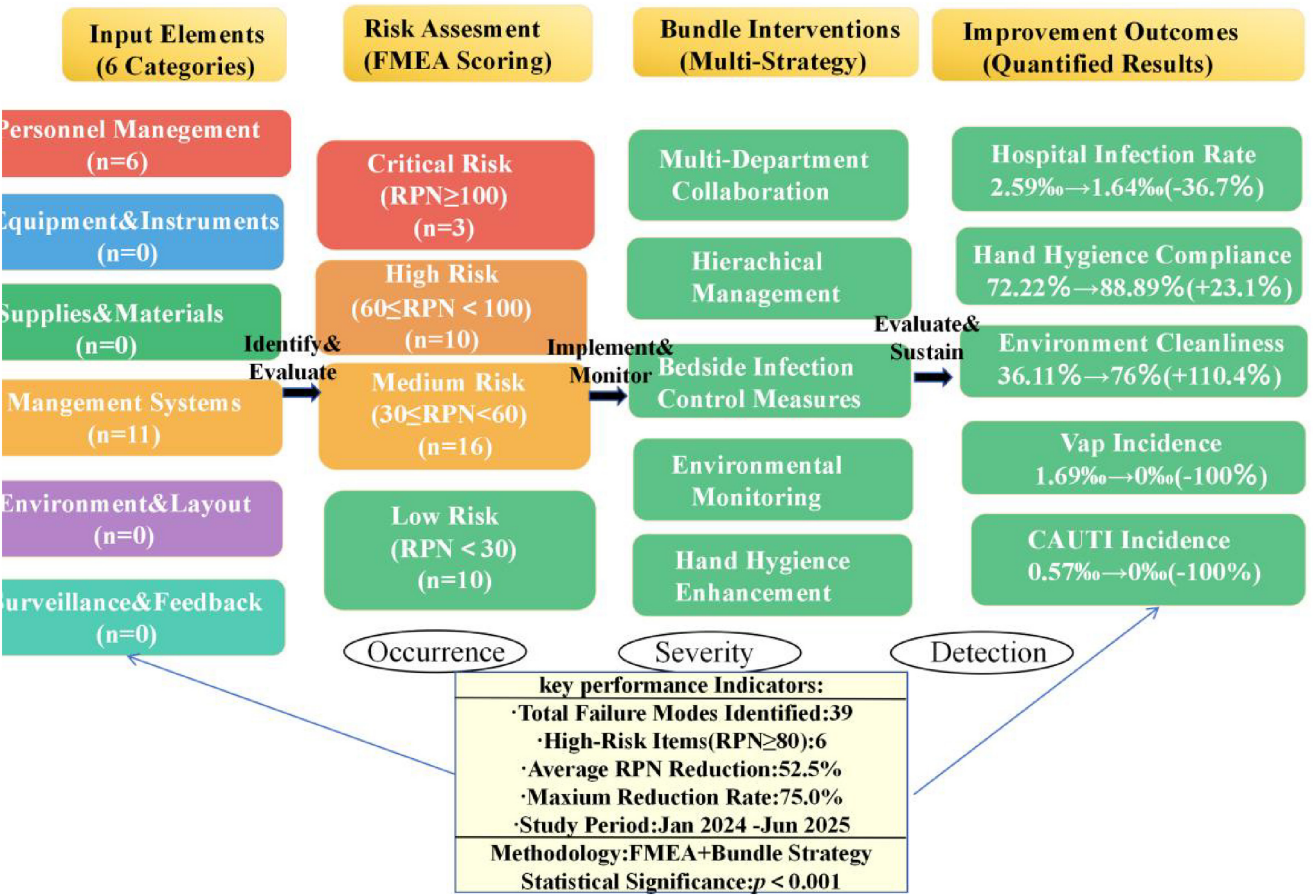
Flowchart of hospital infection management and control.

Using literature review, brainstorming, and considering the actual working conditions of the ICU, potential risk factors for ICU-acquired infections were systematically identified and analyzed from the six elements: Man, Machine, Material, Method, Environment, and Measurement (14). A flowchart for ICU infection management and control was developed (see Figure 1), enabling comprehensive identification of risk indicators related to hospital infection management.

#### 2.2.3 Identification of potential failure modes and analysis of causes

Each sub-process within the ICU hospital-acquired infection management workflow was thoroughly examined to systematically identify potential failure modes. An in-depth root cause analysis was conducted for each identified failure mode.

#### 2.2.4 Risk quantification analysis

**FIGURE 2:**
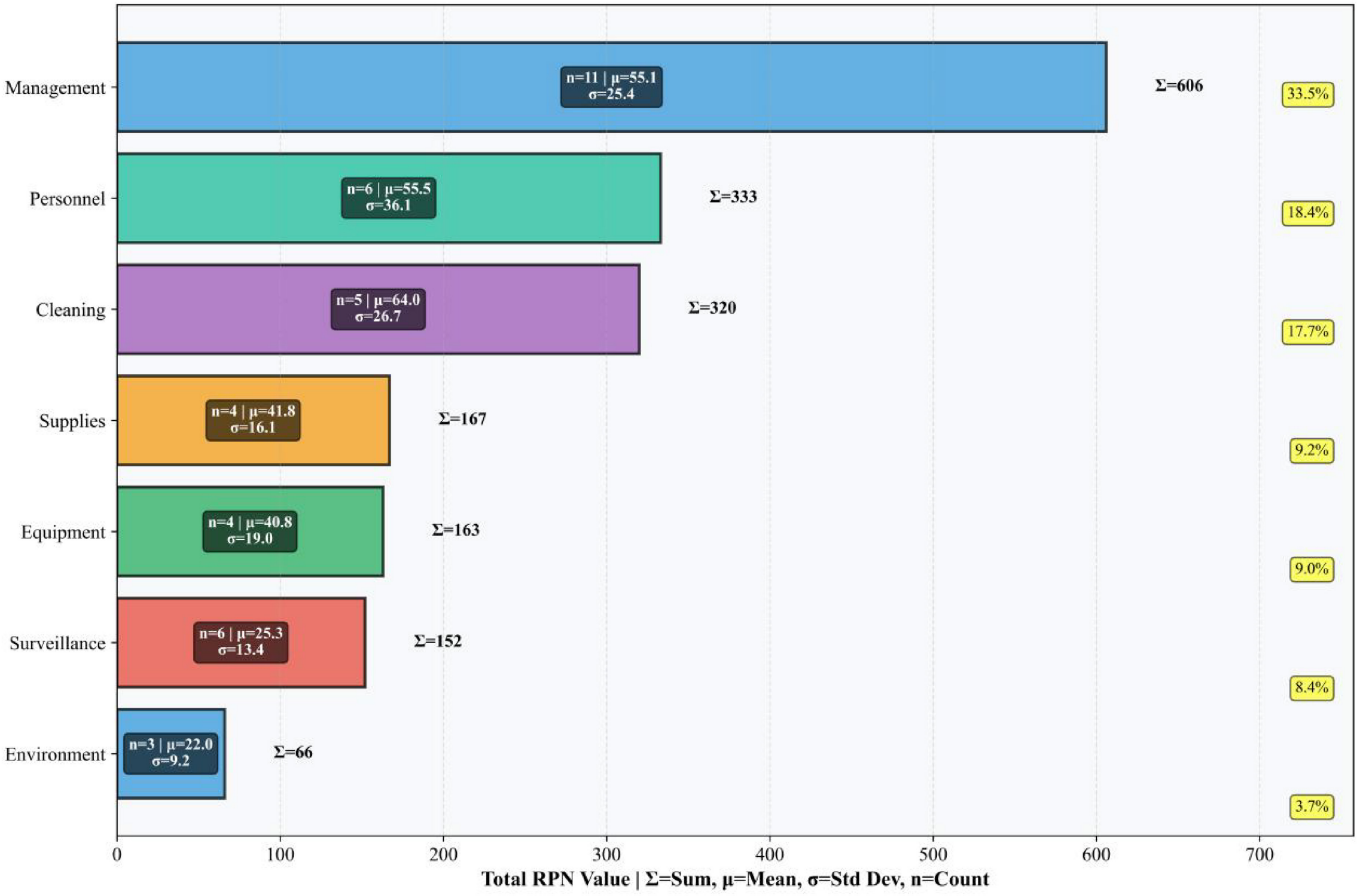
Six-category risk distribution analysis total risk, mean, standard deviation and sample size.

**FIGURE 3:**
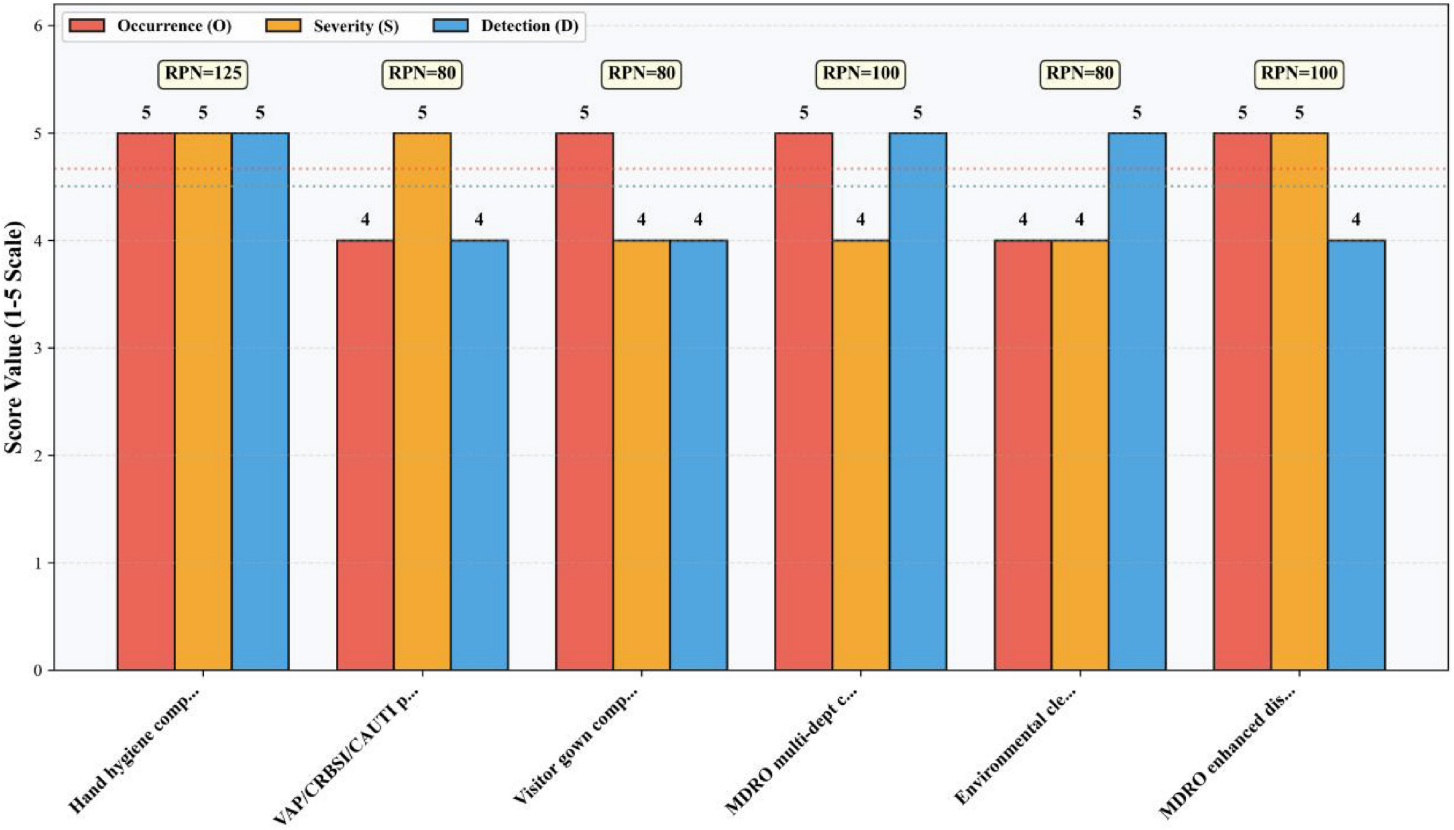
O-S-D three-dimensional score comparison high-risk items (RPN ≥ 80) with total RPN display.

The identified risk factors were scored on a 5-point scale across three dimensions: Occurrence (O), Severity (S), and Detectability (D). The FMEA scoring criteria for Occurrence, Severity, and Detectability are detailed in Tables 1–3, respectively. A quantitative assessment of the infection risk factors was performed using the Risk Priority Number (RPN), calculated as RPN = O × S × D. High-risk failure modes were determined based on the calculated RPN values. The potential failure modes for ICU-acquired infections and their corresponding RPN values are provided in Supplementary File 1 and Figures 2, 3.

**TABLE 1:** FMEA occurrence scoring criteria.

Likelihood (O)	Probability of occurrence	Score
Very high, almost certain	≥ 1/2	5
High, frequent	1/8	4
Moderate, occasional	1/80	3
Low, uncommon	1/1,000	2
Very low, remote	≤ 1/10,000	1

**TABLE 2:** FMEA severity scoring criteria.

Severity (S)	Criteria	Score
Catastrophic	Results in patient death	5
Major	Results in permanent harm to patient	4
Moderate	Causes or prolongs hospitalization	3
Minor	Causes minor harm to patient	2
Negligible	No harm, but negatively impacts patient satisfaction	1

**TABLE 3:** FMEA detection scoring criteria.

Detection (D)	Score
Absolute or almost impossible detection	5
Very low or remote likelihood of detection	4
Low or moderate likelihood of detection	3
Moderately high or high likelihood of detection	2
Very high or almost certain detection	1

#### 2.2.5 Formulation of bundled management intervention measures

Through multi-departmental coordination, the responsibilities of each department were clarified. In combination with the hospital’s actual conditions, a multifaceted intervention strategy was implemented, integrating evidence-based infection control measures, education and training, as well as monitoring and evaluation, to collectively act on the prevention and control of HAIs (15, 16). Specific measures included:

(1) Establishment of a Multi-departmental Coordination Mechanism: A multi-departmental working group for ICU HAI prevention and control was formed, comprising representatives from medical, nursing, infection control, pharmacy, laboratory, and logistics departments. This group conducted regular supervisory inspections and provided timely feedback. Regular joint meetings were held to systematically analyze problems encountered in HAI prevention and control work and to propose targeted improvement measures.
(2) Implementation of Stratified Personnel Management: Precise stratified management was carried out for different groups. For medical staff, standardized clinical procedures were reinforced to continuously enhance awareness and capability in HAI prevention and control. Specialized training for environmental services staff was conducted to improve environmental cleaning and disinfection compliance rates and standardize medical waste collection and transportation. Patients were cohorted based on their infection status according to the surveillance definitions of the National Healthcare Safety Network (NHSN) at the Centers for Disease Control and Prevention (CDC). The categories were defined as follows: (a) “Infected”—patients meeting NHSN criteria for a specific infection type (e.g., VAP, CLABSI, CAUTI); (b) “Suspected infection”—patients presenting with clinical signs or symptoms suggestive of infection (e.g., fever, leukocytosis, purulent secretions) but lacking confirmatory microbiological or radiographic evidence; (c) “Non-infected”—patients without any clinical or microbiological evidence of infection. Management of visitors and visitation procedures was strengthened, strictly limiting the number of visitors and visitation duration.

Management of visitors and visitation procedures was strengthened, strictly limiting the number of visitors and visitation duration. The specific visitation policy implemented was as follows: (a) each patient was permitted a maximum of one designated visitor per day; (b) visitation duration was limited to 30 min per session, with a maximum of one session per day; (c) visitors were required to perform hand hygiene upon entry and exit, wear appropriate personal protective equipment (e.g., surgical masks, isolation gowns if indicated), and remain within the patient’s designated area; (d) visitors with symptoms of respiratory infection, fever, or known communicable diseases were prohibited from entry. Operationalization was achieved through (i) signage at the ICU entrance outlining visitation rules; (ii) training of security and nursing staff to enforce the policy; (iii) issuance of visitor passes with time-stamped entry and exit logs. Adherence was monitored through (i) daily audits by nursing shift leaders; (ii) weekly random spot checks by infection control personnel; (iii) documentation review of visitor logs and hand hygiene compliance observations.

(3) Reinforcement of Device-Associated Infection Prevention and Control: Building upon routine preventive measures for VAP, CRBSI, and CAUTI as per clinical practice guidelines, a focused effort was made to evaluate the execution of core infection prevention measures at the bedside to ensure their proper implementation.

The following clinical practice guidelines were referenced:

- For VAP: Guidelines for the Prevention of Ventilator-Associated Pneumonia issued by the Healthcare Infection Control Practices Advisory Committee (HICPAC)/Centers for Disease Control and Prevention (CDC) (17).
- For CLABSI/CRBSI: The Society for Healthcare Epidemiology of America (SHEA)/Infectious Diseases Society of America (IDSA) Strategies to Prevent Central Line-Associated Bloodstream Infections in Acute Care Hospitals (18).
- For CAUTI: SHEA/IDSA Strategies to Prevent Catheter-Associated Urinary Tract Infections in Acute Care Hospitals (19).

The bundled preventive measures included:

- VAP prevention bundle: (a) head-of-bed elevation (30°–45°); (b) daily sedation interruption and assessment for extubation readiness; (c) oral care with chlorhexidine (twice daily); (d) prevention of ventilator circuit condensate accumulation; (e) strict hand hygiene before and after airway manipulation.
- CLABSI prevention bundle: (a) use of a central line insertion checklist; (b) maximal sterile barrier precautions during insertion (cap, mask, sterile gown, sterile gloves, large sterile drape); (c) chlorhexidine skin antisepsis prior to insertion; (d) daily assessment of line necessity with prompt removal when no longer indicated; (e) substitution of central lines with peripheral catheters whenever feasible.
- CAUTI prevention bundle: (a) insertion only for appropriate indications; (b) strict aseptic technique during insertion; (c) maintenance of a closed drainage system; (d) daily assessment of catheter necessity with prompt removal; (e) securement of the catheter to prevent movement and urethral traction.

Adherence to these measures was evaluated at the bedside through:

- Direct observation: Infection control nurses conducted unannounced bedside audits using standardized checklists for each bundle component, with a minimum of 10 observations per month per device type.
- Documentation review: Weekly review of medical records to assess documentation of daily necessity assessments (e.g., daily sedation interruption for VAP, daily line/catheter necessity for CLABSI/CAUTI).
- Feedback mechanism: Audit results were summarized monthly and shared with ICU staff during multidisciplinary meetings, with corrective actions implemented for any identified gaps (e.g., re-education for specific staff, reinforcement of checklist use).

(4) Implementation of a Hand Hygiene Improvement Program: Various methods were employed to continuously improve hand hygiene compliance, including ensuring convenient access to hand hygiene facilities, conducting hand hygiene training and assessments, and establishing a monitoring and feedback mechanism for hand hygiene compliance.
(5) Strengthening Multidrug-Resistant Organism (MDRO) Management: A Standard Operating Procedure (SOP) for MDRO management was developed. Placement and isolation measures for patients with MDROs were strictly implemented, with early isolation initiated for high-risk populations. Decolonization therapy was also implemented to reduce HAI incidence.
(6) Enhancing Environmental Cleaning and Disinfection Quality: Measures were taken to ensure the thorough implementation of environmental cleaning and disinfection protocols. Environmental microbial monitoring was conducted quarterly. The fluorescent marker method was used periodically to assess the quality of environmental cleaning and disinfection, facilitating prompt identification and rectification of issues.

### 2.3 Statistical methods

Statistical analysis was performed using SPSS software (version 26.0). This was a before-after cohort study with independent samples, meaning that different patients were enrolled in the pre-implementation period (January–December 2024) and the post-implementation period (January–June 2025), with no patient overlap between the two periods. Categorical data were presented as numbers (percentages). Comparisons between the two independent periods were performed using the Chi-square test or Fisher’s exact test for categorical variables, and the Mann-Whitney U test for continuous variables as appropriate. A *P*< 0.05 was considered statistically significant.

## 3 Results

### 3.1 Comparison of baseline characteristics

Baseline characteristics were compared between the independent patient samples from the pre-implementation period (January–December 2024, *n* = 2,975) and the post-implementation period (January–June 2025, *n* = 1,522) using data extracted from electronic medical records. No statistically significant differences were observed between the two periods in terms of age (*t* = -1.234, *P* = 0.217), sex (χ^2^ = 0.312, *P* = 0.576), primary diagnosis distribution (χ^2^ = 2.456, *P* = 0.873), APACHE II score (*t* = -1.156, *P* = 0.248), comorbidities (diabetes: χ^2^ = 0.001, *P* = 0.978; chronic kidney disease: χ^2^ = 0.001, *P* = 0.975; chronic heart failure: χ^2^ = 0.003, *P* = 0.954), or ICU length of stay (Z = -1.567, *P* = 0.117). These results confirm that the two independent cohorts were comparable at baseline. Details are shown in Table 4.

**TABLE 4:** Comparison of baseline characteristics between pre-implementation and post-implementation periods.

Characteristic	Pre-implementation (Jan–Dec 2024, *n* = 2,975)	Post-implementation (Jan–Jun 2025, *n* = 1,522)	Statistical test	*P*-value
Age (years)	59.8 ± 15.2	60.4 ± 14.9	*t* = -1.234	0.217
Sex, male, n (%)	1,389 (46.7)	724 (47.6)	χ^2^ = 0.312	0.576
Primary diagnosis, n (%)			χ^2^ = 2.456	0.873
- Lung cancer	892 (30.0)	456 (30.0)		
- Colorectal cancer	654 (22.0)	334 (21.9)		
- Gastric cancer	445 (15.0)	228 (15.0)		
- Other malignancies	984 (33.1)	504 (33.1)		
APACHE II score	18.5 ± 5.2	18.7 ± 5.1	*t* = -1.156	0.248
Comorbidities, n (%)				
- Diabetes mellitus	654 (22.0)	335 (22.0)	χ^2^ = 0.001	0.978
- Chronic kidney disease	297 (10.0)	152 (10.0)	χ^2^ = 0.001	0.975
- Chronic heart failure	446 (15.0)	229 (15.0)	χ^2^ = 0.003	0.954
ICU length of stay (days), median (IQR)	3.4 (2.0–6.2)	3.2 (1.9–5.9)	*Z* = -1.567	0.117

No patient was included in both periods. All baseline characteristics were extracted from electronic medical records for the full populations in each period. SD, standard deviation; IQR, interquartile range; APACHE II, Acute Physiology and Chronic Health Evaluation II.

### 3.2 Changes in RPN values for high-risk failure modes

**FIGURE 4:**
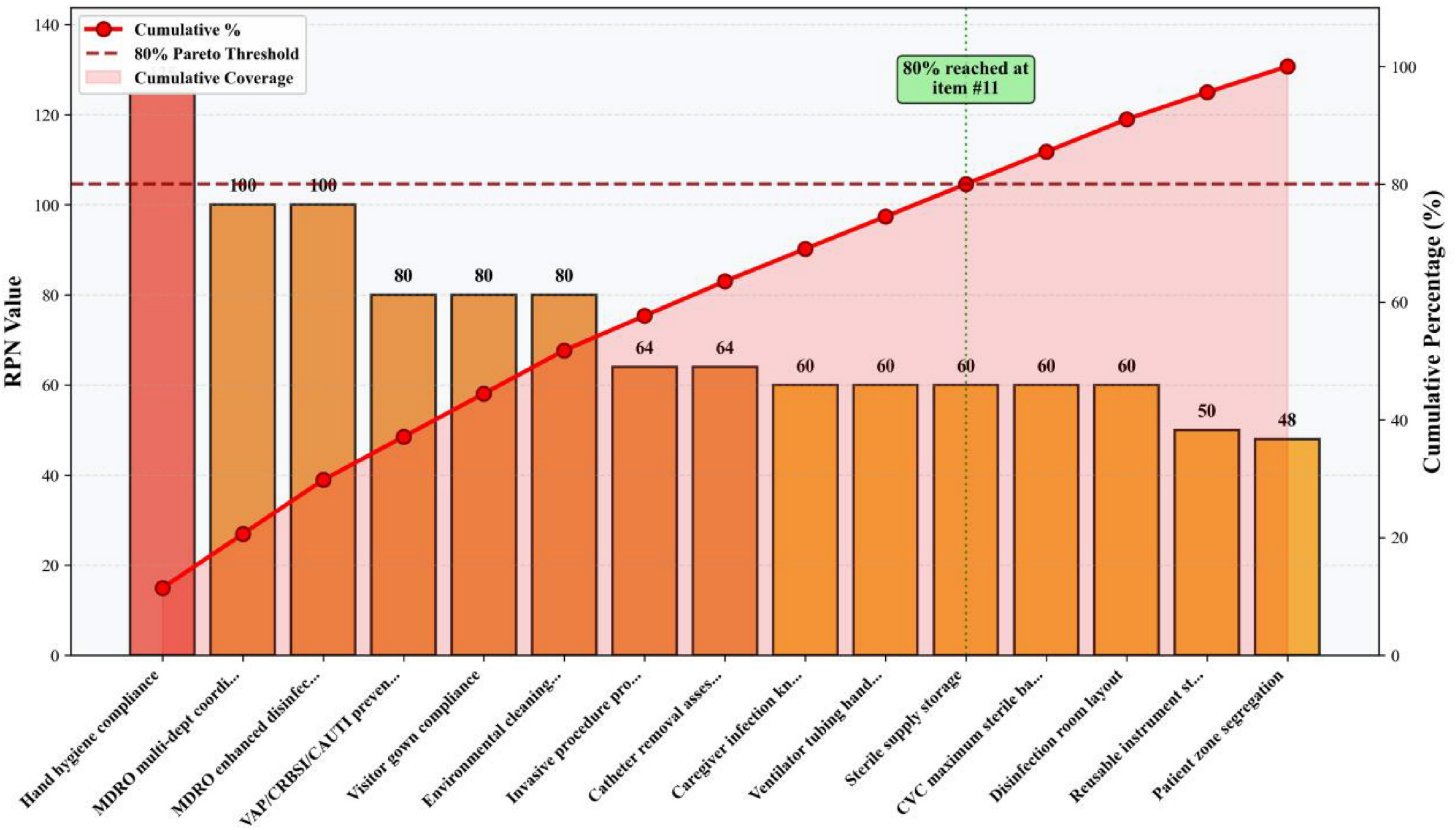
Pareto analysis of failure modes (Top 15).

**FIGURE 5:**
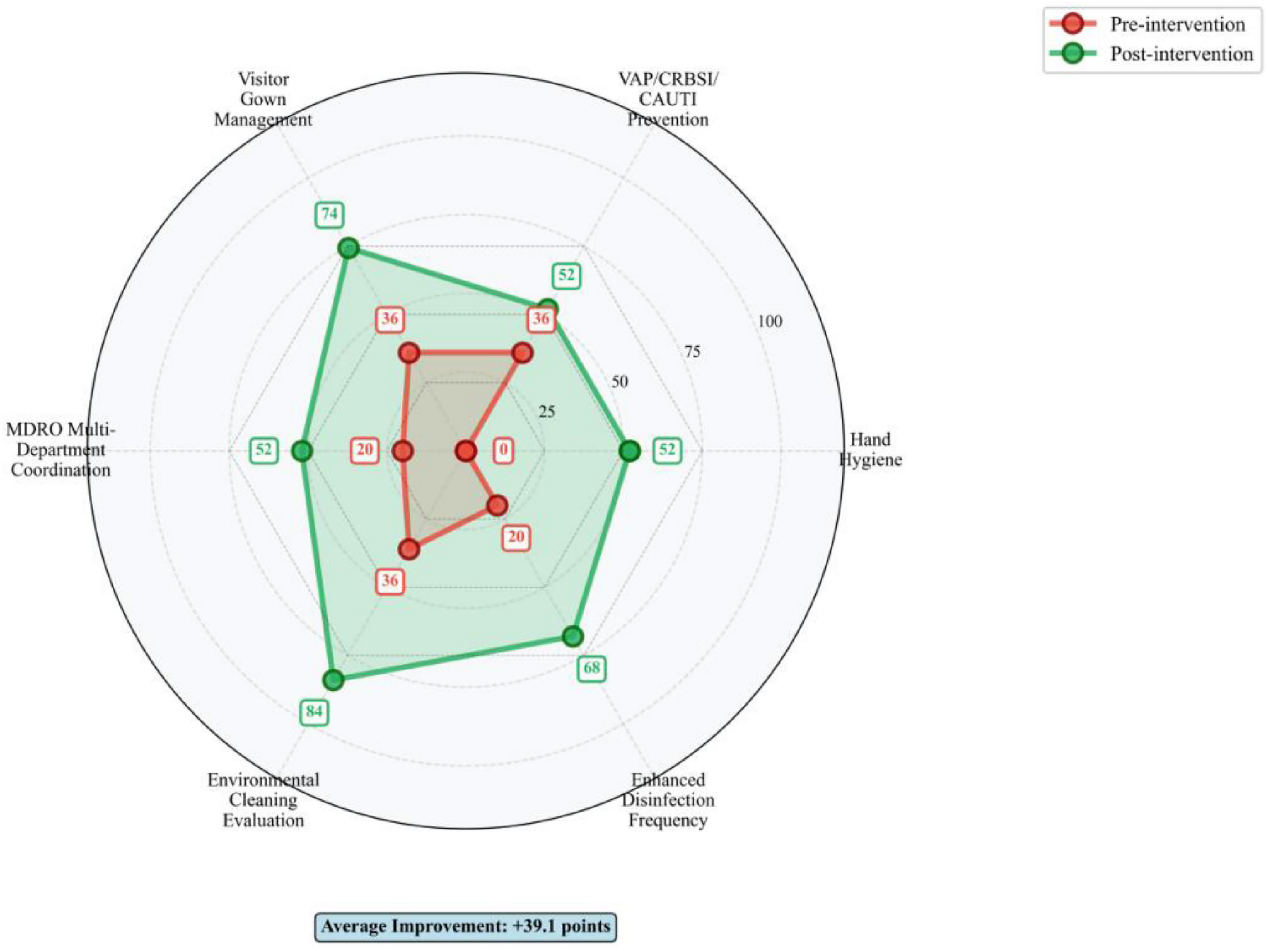
Multi-dimensional improvement radar chart.

**FIGURE 6:**
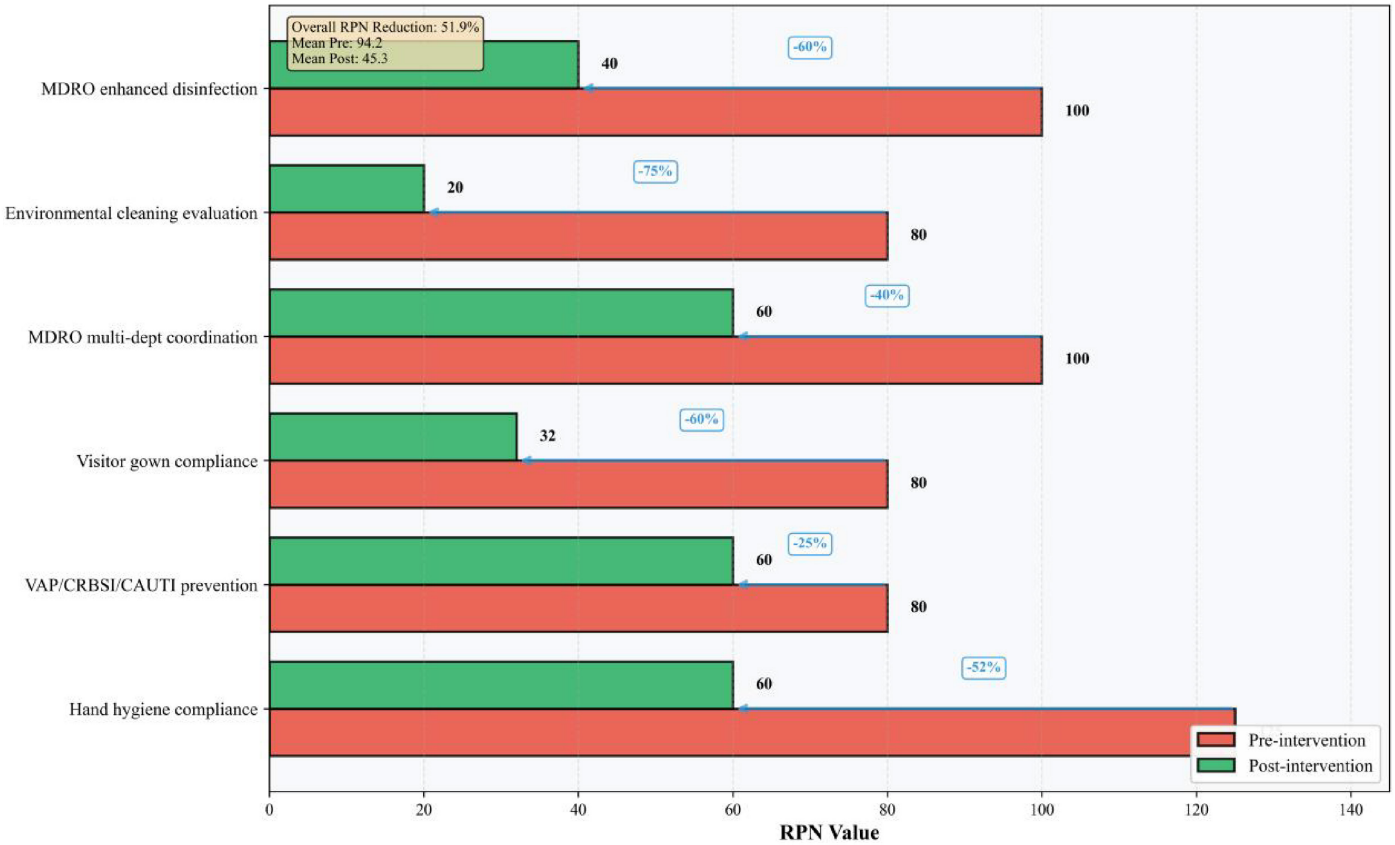
High-risk failure modes-RPN comparison pre vs. post intervention with reduction rate.

High-risk failure modes were determined according to the Pareto principle (“80/20 rule”). Following the implementation of bundled management measures targeting these high-risk failure modes, the RPN values for ICU-acquired infection high-risk failure modes decreased significantly. Details are presented in Table 5 and Figures 4–6, the corresponding visualization chart.

**TABLE 5:** Comparison of RPN for high-risk process steps before and after FMEA implementation.

Potential failure mode	Original RPN score	Post-implementation RPN score	Reduction (%)
1. Suboptimal hand hygiene compliance among healthcare workers	125	90	28.0
2. Ineffective multi-departmental coordination for MDRO prevention and control	100	45	55.0
3. Failure to implement increased disinfection frequency for MDRO patients	100	50	50.0
4. Failure to promptly evaluate the effectiveness of environmental cleaning/disinfection	80	20	75.0
5. Poor implementation of dedicated bed-specific visitor gowns and post-visit cleaning/disinfection	80	30	62.5
6. Cleaning staff fail to strictly implement “one cloth per bed” and zonal use of tools	48	20	58.3

### 3.3 Comparison of hospital-acquired infection incidence

Following the implementation of the FMEA-driven bundled management measures, the incidence of HAIs among ICU patients decreased significantly. The infection rates were calculated based on a total of 2,975 patient-monitored days in the pre-implementation period (2024) and 1,522 patient-monitored days in the post-implementation period (January–June 2025), with 77 and 25 infection cases, respectively. The HAI incidence rate from January to June 2025 was 1.6%, which was significantly lower than the 2.6% rate in 2024 (χ^2^ = 4.062, *P* < 0.05). Other related metrics, including the HAI case rate, daily incidence rate, daily case rate, and adjusted daily case rate, all showed varying degrees of reduction. Details are presented in Table 6.

**TABLE 6:** HAI incidence among ICU patients before and after implementation.

Year/period	Patients monitored (n)	Infection cases (n)	HAI incidence rate (%)	Infection episodes (n)	Infection episode rate (%)	Total patient-days	Average severity of illness	Daily incidence rate (‰)	Daily episode rate (‰)	Adjusted daily episode rate (‰)
2024 (Pre-implementation)	2,975	77	2.6	82	2.8	10,117	3.13	7.6	8.1	2.6
Jan-Jun 2025 (post-implementation)	1,522	25	1.6	26	1.7	4,831	3.11	5.2	5.4	1.7

### 3.4 Comparison of device-associated infection rates

**FIGURE 7:**
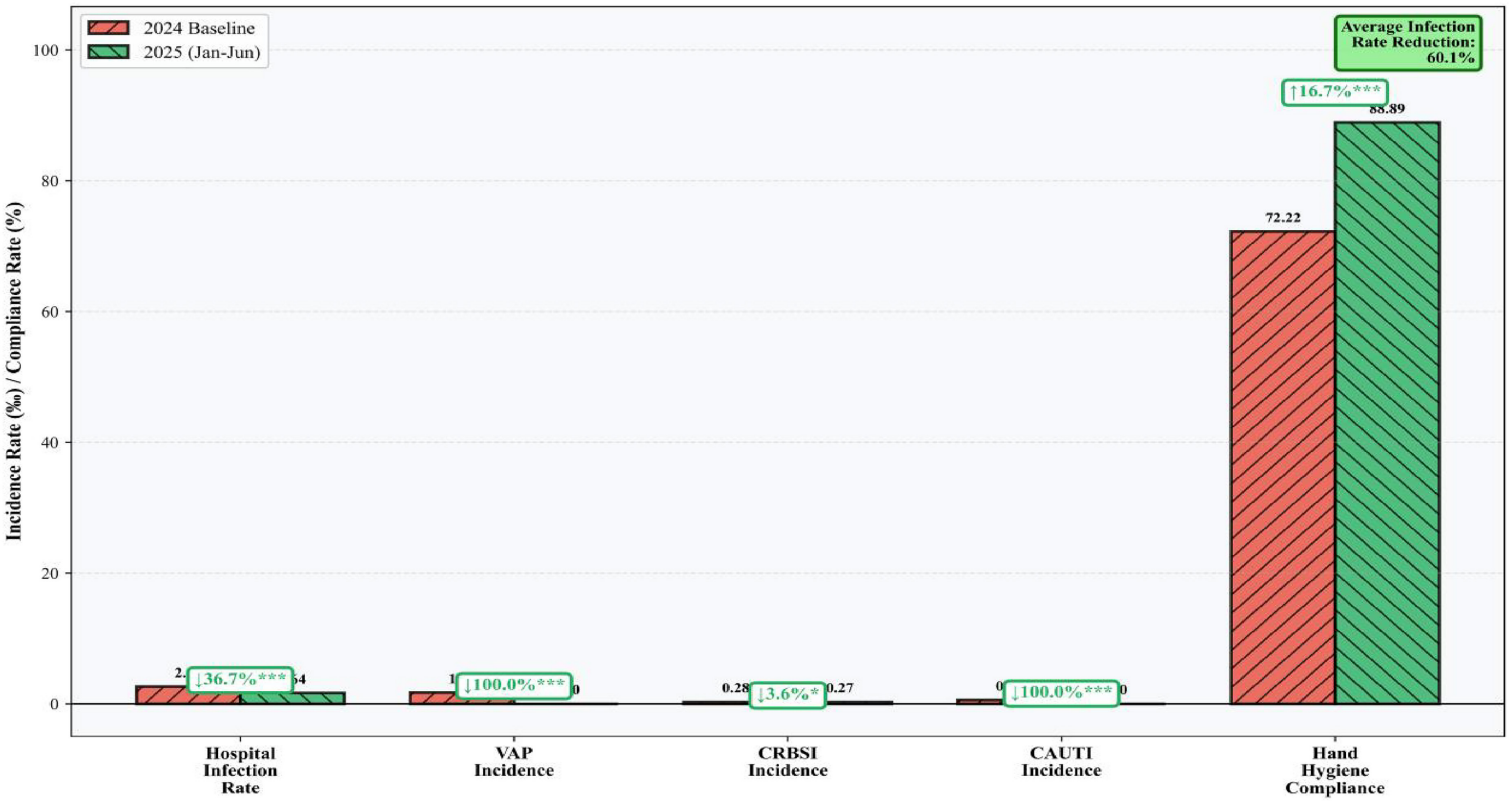
Evaluation of environmental cleaning and disinfection quality.

Following the implementation of bundled management measures, device-associated infection control showed significant efficacy. From January to June 2025, no cases of Ventilator-Associated Pneumonia (VAP) or Catheter-Associated Urinary Tract Infection (CAUTI) occurred, with only one case of Catheter-Related Bloodstream Infection (CRBSI) reported. The details of device-associated infections are presented in Table 7 and Figure 7.

**TABLE 7:** Incidence of device-associated infections in ICU patients before and after implementation.

Time period	Urinary catheter			Central venous catheter			Invasive ventilator		
	No. of infections	Device-days	CAUTI rate (‰)	No. of infections	Device-days	CRBSI rate (‰)	No. of infections	Device-days	VAP rate (‰)
2024	4	6,970	0.57	2	7,154	0.28	3	1,774	1.69
Jan-Jun 2025	0	3,276	0	1	3,706	0.27	0	770	0

### 3.5 Changes in hand hygiene compliance rate

Following the implementation of the FMEA-based bundled management strategy, the hand hygiene compliance rate among ICU healthcare workers increased significantly from 72.2% (52/72) pre-implementation to 88.9% (40/45) post-implementation (χ^2^ = 4.578, *P* < 0.05), representing an improvement of 23.1%.

### 3.6 Evaluation of environmental cleaning and disinfection quality

The quality of environmental cleaning and disinfection was assessed using the fluorescent marker method. The 24-h removal rate of markers rose from 36.1% before implementation to 76.0% after, marking a significant improvement of 110.5% and indicating a substantial enhancement in environmental cleaning and disinfection quality.

## 4 Discussion

### 4.1 Specificity and challenges of HAI prevention and control in the ICU of a specialized oncology hospital

In contemporary medical practice, with the continuous advancement of oncology diagnosis and treatment systems, the complex profile of ICU patients in specialized oncology hospitals—characterized by immunosuppression due to malignancy itself, intensive radiotherapy/chemotherapy, frequent invasive procedures, and antibiotic misuse—has led to increasingly prominent issues of multidrug-resistant organism colonization and infection (20). Therefore, effective HAI prevention and control is a core component influencing patient survival, healthcare quality, and resource utilization. Traditional HAI prevention and control models often rely on passive surveillance and experiential management, lacking systematic risk identification and prospective prevention mechanisms, making it difficult to meet the complex infection control demands of the ICU in specialized oncology hospitals.

### 4.2 Application value of FMEA in HAI risk management

As a prospective, multidisciplinary risk assessment tool, FMEA offers significant advantages in quantifiability and proactivity (21). Its utility has been demonstrated across various infection prevention domains, including multidrug-resistant organism control (22), endoscope cleaning quality improvement (23, 24), surgical site infection prevention (25, 26), and central line-associated bloodstream infection control (27). In our study, FMEA enabled the systematic identification of six high-risk failure modes (RPN ≥ 80), which encompassed deficits in hand hygiene, device-associated infection prevention bundle adherence, visitor management, multi-departmental coordination, environmental cleaning evaluation, and disinfection practices. By quantifying risk priorities, FMEA facilitated a shift from reactive problem-solving to proactive, targeted intervention (28), effectively pinpointing the most vulnerable points in the infection control process.

### 4.3 Comprehensive intervention effects of the bundled management model

The integration of FMEA with bundled management enabled a systematic, multi-layered intervention approach. The multi-departmental coordination mechanism—facilitated through regular joint meetings—ensured timely feedback and iterative refinement of infection prevention protocols. Stratified training tailored to the roles of physicians, nurses, environmental services staff, and nursing assistants enhanced infection prevention competency across disciplines.

The intervention yielded substantial clinical improvements. Overall HAI incidence declined by 36.7% (from 2.59 to 1.64%), a reduction that aligns with the magnitude of effect reported in other FMEA-based quality improvement studies (12, 18). Notably, CAUTI and VAP rates dropped to zero, and hand hygiene compliance increased by 23.1%. The marked improvement in environmental cleaning quality (110.5% increase in fluorescent marker removal) suggests that addressing previously overlooked environmental contamination pathways was a key driver of infection reduction. These findings underscore the value of combining prospective risk assessment (FMEA) with bundled, multi-dimensional interventions to achieve synergistic effects that surpass those of isolated measures (15, 16).

### 4.4 Innovation and limitations of the study

The innovative aspects of this study are as follows: it is the first to organically combine FMEA with bundled management and apply it to the entire HAI prevention and control process in the ICU of a specialized oncology hospital; it systematically identified 47 potential failure modes using the “Man, Machine, Material, Method, Environment, Measurement” analysis framework; and through RPN quantitative scoring, it precisely screened six high-risk failure modes, achieving a shift from experiential management to precise prevention and control.

However, this study also has certain limitations. First, the study duration was relatively short, and long-term effects require further observation. Second, as a single-center study, the generalizability of the results needs validation through multi-center research. Third, the baseline characteristic comparison was performed on a relatively small subset of patients (*n* = 82 pre-implementation, *n* = 26 post-implementation). Consequently, the absence of statistically significant differences between the two periods should be interpreted with caution, as it may reflect limited statistical power rather than true comparability. Although the demographic characteristics appeared numerically similar, we cannot exclude the possibility of unmeasured confounding due to the small sample size for baseline analysis. Fourth, observation of some process indicators (e.g., hand hygiene compliance rate) may be subject to the Hawthorne effect. Future research should extend the observation period, increase sample size for baseline comparisons, and explore the application of information technology in the continuous improvement of FMEA.

### 4.5 Clinical practice significance and promotion value

The findings of this study offer important implications for clinical practice. First, the FMEA method can assist healthcare institutions in identifying high-risk vulnerabilities within their infection prevention and control systems, facilitating a shift from passive response to proactive prevention. Second, the bundled management strategy, through multi-dimensional collaborative interventions, can generate synergistic effects, significantly enhancing prevention and control outcomes. Third, the combination of multi-departmental coordination and stratified management ensures the precise implementation and continuous improvement of intervention measures.

## 5 Conclusion

In summary, the approach based on FMEA combined with bundled management measures can scientifically and accurately identify risk points within the ICU HAI prevention and control process and implement targeted interventions, achieving effective integration of risk management and quality improvement. Through prospective risk quantification assessment and multi-departmental collaborative intervention, this study significantly reduced the incidence of HAIs and device-associated infections while improving hand hygiene compliance and environmental disinfection quality. It provides an evidence-based practice paradigm for the precise prevention and control of HAIs in the ICU of specialized oncology hospitals. This management methodology and improvement measures hold significant value for promotion and application in reducing ICU HAI incidence and are worthy of reference and adoption by similar healthcare institutions.

## Data Availability

The raw data supporting the conclusions of this article will be made available by the authors, without undue reservation.
