# Supplementary material for: Application of failure mode and effects analysis combined with bundled management measures in reducing ICU-acquired infections
Source: Front Med (Lausanne). 2026 May 22;13:1801802. doi: 10.3389/fmed.2026.1801802 (PMC13237694; doi:10.3389/fmed.2026.1801802)
Supplement: Supplementary file 1 [file Table_1.doc]

***Table 4 Potential Failure Modes and Risk Priority Numbers for ICU-Acquired Infections***

| Potential Failure Mode | Potential Failure Cause | Occurrence (O) | Severity (S) | Detection (D) | Risk Priority Number (RPN) |
| --- | --- | --- | --- | --- | --- |
| ****Inadequate Personnel Management**** | 1. Suboptimal hand hygiene compliance among healthcare workers | 5 | 5 | 5 | 125 |
|  | 2. Poor aseptic awareness and non-standardized practices among staff | 4 | 3 | 3 | 36 |
|  | 3. Insufficient knowledge of infection control among nursing assistants | 5 | 3 | 4 | 60 |
|  | 4. Patient-related factors (e.g., comorbidities, compromised immunity) | 4 | 2 | 3 | 24 |
|  | 5. Failure to implement zoning placement for different patient types | 3 | 4 | 4 | 48 |
|  | 6. Inadequate visitor management (e.g., failure to limit visitor numbers) | 5 | 2 | 4 | 40 |
| ****Non-standardized Use of Medical Equipment/Devices**** | 1. Improper handling of ventilator circuits and condensate | 4 | 5 | 3 | 60 |
|  | 2. Non-standardized invasive procedures (e.g., endotracheal intubation) | 4 | 5 | 2 | 40 |
|  | 3. Inadequate implementation of device cleaning and disinfection | 3 | 4 | 4 | 48 |
|  | 4. Lack of regular equipment maintenance and servicing | 1 | 3 | 5 | 15 |
| ****Inadequate Management of Medical Supplies**** | 1. Improper storage of single-use sterile items (damage, expiration) | 3 | 4 | 5 | 60 |
|  | 2. Substandard cleaning, disinfection, or sterilization of reusable instruments | 2 | 5 | 5 | 50 |
|  | 3. Inadequate preparation of personal protective equipment (PPE) | 1 | 5 | 5 | 25 |
|  | 4. Inappropriate use of antimicrobial agents | 2 | 4 | 4 | 32 |
| ****Imperfect Management Systems & Operational Procedures**** | 1. Inadequate establishment/function of the infection control organizational structure | 3 | 4 | 3 | 36 |
|  | 2. Failure to regularly review and update infection control policies/procedures | 2 | 3 | 3 | 18 |
|  | 3. Insufficient supervision and enforcement of control measures | 3 | 3 | 4 | 36 |
|  | 4. Ineffective implementation of measures to prevent VAP, CRBSI, CAUTI | 4 | 5 | 4 | 80 |
|  | 5. Non-standardized invasive procedures (e.g., endotracheal intubation) | 4 | 4 | 4 | 64 |
|  | 6. Delayed assessment for removal of indwelling catheters (venous/urinary) or ventilator, increasing infection risk | 4 | 4 | 4 | 64 |
|  | 7. Failure to adhere to maximal sterile barrier precautions during central venous catheter insertion | 4 | 3 | 5 | 60 |
|  | 8. Non-standardized catheter maintenance (dressing changes, line flushing) | 2 | 4 | 4 | 32 |
|  | 9. Poor implementation of dedicated bed-specific visitor gowns and post-visit cleaning/disinfection | 5 | 4 | 4 | 80 |
|  | 10. Staff training is perfunctory, with no follow-up on effectiveness | 4 | 3 | 3 | 36 |
|  | 11. Ineffective multi-departmental coordination for MDRO prevention and control | 5 | 4 | 5 | 100 |
| ****Suboptimal Environmental Layout**** | 1. Unreasonable layout in some areas leading to cross-contamination | 2 | 3 | 4 | 24 |
|  | 2. Poor ventilation | 1 | 3 | 4 | 12 |
|  | 3. Insufficient isolation room capacity | 2 | 3 | 5 | 30 |
| ****Substandard Environmental Cleaning & Disinfection**** | 1. Poor layout/ventilation in cleaning rooms; incomplete drying of cleaning tools | 4 | 3 | 5 | 60 |
|  | 2. Failure to promptly evaluate the effectiveness of environmental cleaning/disinfection | 4 | 4 | 5 | 80 |
|  | 3. Cleaning staff fail to strictly implement "one cloth per bed" and zonal use of tools | 3 | 4 | 4 | 48 |
|  | 4. Non-standard preparation of disinfectants (incorrect concentration) | 2 | 4 | 4 | 32 |
|  | 5. Failure to implement increased disinfection frequency for MDRO patients | 5 | 5 | 4 | 100 |
| ****Delayed Infection Surveillance & Feedback**** | 1. Inadequate competency of dedicated staff; inaccurate surveillance data | 1 | 4 | 3 | 12 |
|  | 2. Failure to timely summarize/analyze surveillance data to identify risks | 3 | 3 | 3 | 27 |
|  | 3. Underutilization of informatization for infection early warning | 2 | 4 | 4 | 32 |
|  | 4. Insufficient intensity in process management for targeted surveillance | 4 | 3 | 4 | 48 |
|  | 5. Ineffective emergency drills | 2 | 3 | 3 | 18 |
|  | 6. Inadequate capability to identify outbreak risks | 1 | 5 | 3 | 15 |

***Abbreviations:***VAP: Ventilator-Associated Pneumonia; CRBSI: Catheter-Related Bloodstream Infection; CAUTI: Catheter-Associated Urinary Tract Infection; MDRO: Multidrug-Resistant Organism.
